# Echocardiographic Evaluation of Transitional Circulation for the Neonatologists

**DOI:** 10.3389/fped.2018.00140

**Published:** 2018-05-15

**Authors:** Yogen Singh, Cécile Tissot

**Affiliations:** ^1^Consultant in Neonatology and Pediatric Cardiology, Cambridge University Hospitals NHS Foundation Trust, Cambridge, United Kingdom; ^2^School of Clinical Medicine, University of Cambridge, Cambridge, United Kingdom; ^3^Pediatric Cardiologist, Centre de Pediatrie, Clinique des Grangettes, Chêne-Bougeries, Geneva

**Keywords:** transitional circulation, adverse adaptation of transitional circulation, persistent pulmonary hypertension, hemodynamic assessment, fetal to neonatal transition, patent ductus arteriosus, PDA, PPHN

## Abstract

The hemodynamic changes during the first few breaths after birth are probably the most significant and drastic adaptation in the human life. These changes are critical for a smooth transition of fetal to neonatal circulation. With the cord clamping, lungs take over as the source of oxygenation from placenta. A smooth transition of circulation is a complex mechanism and primarily depends upon the drop in pulmonary vascular resistance (PVR) and increase in systemic vascular resistance (SVR). Understanding the normal transition physiology and the adverse adaptation is of utmost importance to the clinicians looking after neonates. It may have a significant influence on the presentation of congenital heart defects (CHDs) in infants. Bedside echocardiography may help in understanding the transition physiology, especially the hemodynamic changes and shunting across ductus arteriosus and foramen ovale, and it may play an important role in making judicious clinical decisions based upon the altered physiology.

## Introduction

The transition from fetal to the neonatal circulation is a complex process. In fact, the hemodynamic changes during the first few breaths after birth are probably the most significant and drastic adaptation in the human life. Following lung expansion there is a significant decrease in pulmonary vascular resistance (PVR), and removal of the placenta results in an increase in systemic vascular resistance (SVR) (1–4). In majority of the term infants, this is a smooth process and cardiovascular transition is accomplished without any difficulty. However, some infants may have adverse adaptation of the transitional circulation. The conditions leading to poor expansion of lungs, the factors affecting PVR or SVR, and the timing of cord clamping may affect the cardiovascular transition, and hence the etiology is heterogeneous (5–9).

Prematurity has major impact on the transition from fetus to neonatal circulation. The persistence of fetal shunts, high PVR due to increased incidence of respiratory distress syndrome, increased need of mechanical ventilatory support, immature myocardium and increased incidence of metabolic acidosis in the premature infants have major impact on the transition from fetus to neonatal circulation (5, 8, 9).

The phenotypical presentation of the adverse adaptation varies immensely—from mild or no symptoms to a critically ill infant with hypotension and cardiogenic shock. To complicate the matter further, transition of circulation may be affected by the resuscitation manoeuvers, maternal condition, sepsis, medications, thermoregulation, and the interventions infants need in the intensive care setting (9).

Therefore, a good understanding of the cardiovascular transition from fetal to neonatal circulation is of utmost importance to the neonatologists and pediatric cardiologists or intensivists caring for the neonates. It may help the clinicians to choose the appropriate intervention in infants with hemodynamic instability; avoid under- or over-treatment in this vulnerable population cohort, especially preterm infants and critically ill infants with persistent pulmonary hypertension (10–13).

The purpose of this article is to review the changes in cardiovascular physiology during transition from fetal to neonatal circulation, impact of the adverse adaptation on hemodynamics, and how bedside echocardiographic evaluation may help in understanding the clinical relevance and management of the adverse adaptation. For the echocardiographic evaluation of specific condition such as hemodynamic evaluation of patent ductus arteriosus (PDA) is discussed in separately under TINEC research topics in Frontiers in Pediatrics.

## Fetal circulation

In an adult, the cardiovascular system is connected in series, hence, the stroke volume of right and left ventricles are equal. The circulatory connections in a fetus are complex and in parallel facilitated by various intracardiac and extracardiac shunting. In a fetus right ventricle receives 65% of the venous return while left ventricle receives about 35% of the total venous return (4, 14, 15). Both ventricles provide systemic blood flow (systemic cardiac output), and of the combined ventricular output around 45% is directed to the placental circulation while only 8–15% reaches to the pulmonary circulation (14, 15). This complex parallel circulatory arrangement in a fetus is possible because of the following fetal channels:

- The placenta allows the fetus to receive oxygenated blood from the maternal circulation. The umbilical arteries bring around 45% of the fetal cardiac output (de-oxygenated blood) to the placenta, and the oxygenated blood is returned to the fetus via umbilical vein. Placenta has a low vascular resistance and hence SVR in fetus is maintained low (2);
- Ductus arteriosus (DA) facilitate diverting most of the right ventricular cardiac output to the descending aorta. The high PVR in lungs and low SVR facilitate right to left shunting via ductus arteriosus smoothly. The patency of ductus arteriosus is primarily maintained by the high prostaglandin levels synthesized from the placenta and within the ductal tissue, and increased nitric oxide content. The animal studies demonstrate that up to 90% cardiac output is shunted away from un-aerated lungs via ductus arteriosus, although in human fetus this proportion is thought to be bit lower (16, 17). Premature closure of the DA has been reported to result in right heart congestion, persistent pulmonary hypertension, or fetal demise (16–18);
- Ductus venosus plays an important role in directing oxygenated blood from the umbilical vein to the inferior vena cava (toward heart and away from portal venous system); and
- The shape of ductus venosus allows oxygenated blood diversion to inferior vena cava, and Eustachian ridge (at the junction of inferior vena cava with right atrium) helps in preferential shunting of oxygenated blood from umbilical vein to left atrium via foramen ovale. This allows more oxygenated blood returning to left ventricle. Hence, coronary arteries and upper part of the body (brain) receive more oxygenated blood than the lower part of the body (2, 17).

## Normal transition from fetal to neonatal circulation: transition physiology

The transition physiology, from fetal to neonatal circulation, is complex and accompanied by many drastic physiological changes in the cardiovascular system soon after birth. In a term infant, a successful cardiovascular transition is accomplished by an increase in SVR resistance following removal placenta and drop in PVR after expansion of lungs (2, 16).

**Figure 1:**
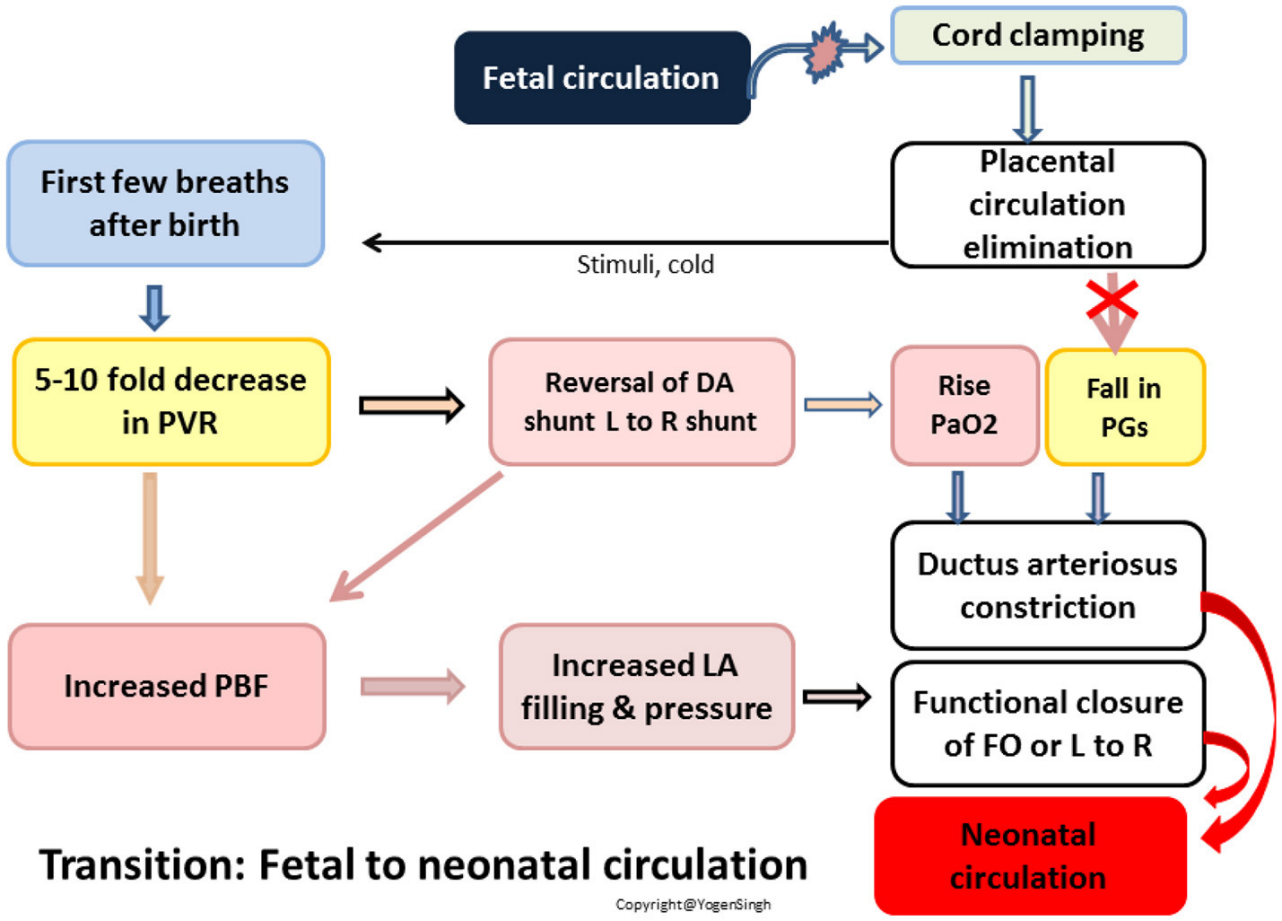
Schematic diagram showing events during transition of circulation: fetal to neonatal circulation. PVR, pulmonary vascular resistance; PBF, pulmonary blood flow; FO, foramen ovale; DA, ductus arteriosus; PaO_2_, arterial partial pressure of oxygen; L, left; R, right; PG, prostaglandin.

The SVR is increased primarily from removal of the low vascular shunt (placenta), but, there are also other important contributing factors such as increase in thromboxane 2 and vasopressin. The drop in PVR is primarily from aeration of lungs, which were filled with amniotic fluid in fetus, and increased production of potent pulmonary arterial vasodilators such as bradykinins, histamine and prostaglandins (19, 20). In a term infant, there is a 5–10-fold drop in PVR soon after normal birth and then it continues to drop over the next 4–6 weeks. This rapid change in SVR and PVR soon after birth is the key for accomplishing successful transition—shunt across ductus arteriosus becomes left to right and there is a rapid increase in pulmonary blood flow (PBF), and hence pulmonary venous return. This results in increase in left atrial pressure and shunt across foramen ovale reverses leading to its functional closure in the first few days after birth. The ductus venosus closes soon after birth (2, 16) (Figure 1).

The mechanism for ductus arteriosus (DA) closure is complex and multifactorial: it is primarily facilitated by the direct vasoconstriction of ductal tissue (smooth muscle) in response to oxygenated blood from left to right shunt via DA, and a drop in prostaglandins after removal of placenta and increased degradation of prostaglandins in the lungs. In a term infant, the functional closure of DA occurs within 24–48 h after birth while anatomical closure may take up to 10–14 days (21).

The closure of fetal shunts (ductus arteriosus, ductus venosus and foramen ovale) and removal of placenta result lead to change in the cardiovascular connections: from parallel in the fetus to in series connections. This accomplishes a successful adaptation of transitional circulation leading to equal left and right ventricular output (Qp:Qs ratio of 1).

The physiological response to transition in preterm infants is even more complex, and a detailed description is beyond the scope of this review article. However, this is briefly discussed later in the section on adverse adaptation.

## Impact of early and delayed cord clamping on cardiovascular physiology

Overall placental-umbilical blood flow increases with gestational age, and it is estimated that placenta receives around 100–125 ml/kg/min (around 40% of the combined biventricular output in fetus) at term). The placenta is thought to hold 30–50% of the fetal circulating volume at any one time. Following immediate cord clamping the estimated mean blood volume of a term infant is approximately 80 ml/Kg (22, 23).

The studies have demonstrated that a term infant may receive an additional 20–35 m//Kg of blood from placental transfusion when clamping of the umbilical cord is delayed for up to 5 min (24). The most of the placental transfusion complete within 3 min and in fact, up to 50% blood is transferred from the placenta within 1 min after birth (23).

In the fetus, the left ventricular preload primarily depends upon the umbilical blood flow via foramen ovale while soon after clamping of the umbilical cord it primarily depends upon the pulmonary venous return. When umbilical cord is clamped before establishment of lung expansion, it may lead to a significant drop in LV preload and systemic cardiac output. This may look less relevant in a well term infant with smooth transition physiology but it becomes more important in the compromised term infant and in the preterm infants—the maintenance of adequate LV preload is essential for sustaining an adequate LV output (16).

There is good evidence showing benefits of delaying cord clamping for 1–3 min. A recent meta-analysis by Fogarty et al., studied 18 randomized controlled trials involving more than 2,300 patients, concluded that delayed cord clamping decrease mortality, improve hemodynamic stability and increase hematocrit values leading to decreased need of blood transfusions in the neonates (25). The other studies have reported decreased need of inotropes, better hemodynamic stability, decreased incidence of intraventricular hemorrhage, decreased incidence of necrotizing enterocolitis, and better long term neurological outcome in the preterm infants (26). However, the latest meta-analysis didn't show any reduction in the incidence of intubation for resuscitation, admission temperature, mechanical ventilation, intraventricular hemorrhage, brain injury, chronic lung disease, PDA, necrotizing enterocolitis, late onset sepsis or retinopathy of prematurity with delayed cord clamping (25). From cardiovascular physiology perspective it's logical to delay the cord clamping for up to 3 min until baby establishes successful lung expansion, and pulmonary venous return and LV preload is established from the increased pulmonary venous return (16).

## Adverse cardiovascular transition

Normal transitional physiology may be altered by many conditions including prematurity, underlying congenital heart defect, infection, perinatal asphyxia, meconium aspiration syndrome (MAS), congenital pneumonia, chest infection, congenital diaphragmatic hernia, and pneumothorax to name a few. While detailed discussion is beyond the scope of this article we will discuss adverse cardiovascular adaptation from persistent high vascular resistance leading to persistent pulmonary hypertension or from increased pulmonary blood from shunting, especially in the premature infants.

**Figure 2:**
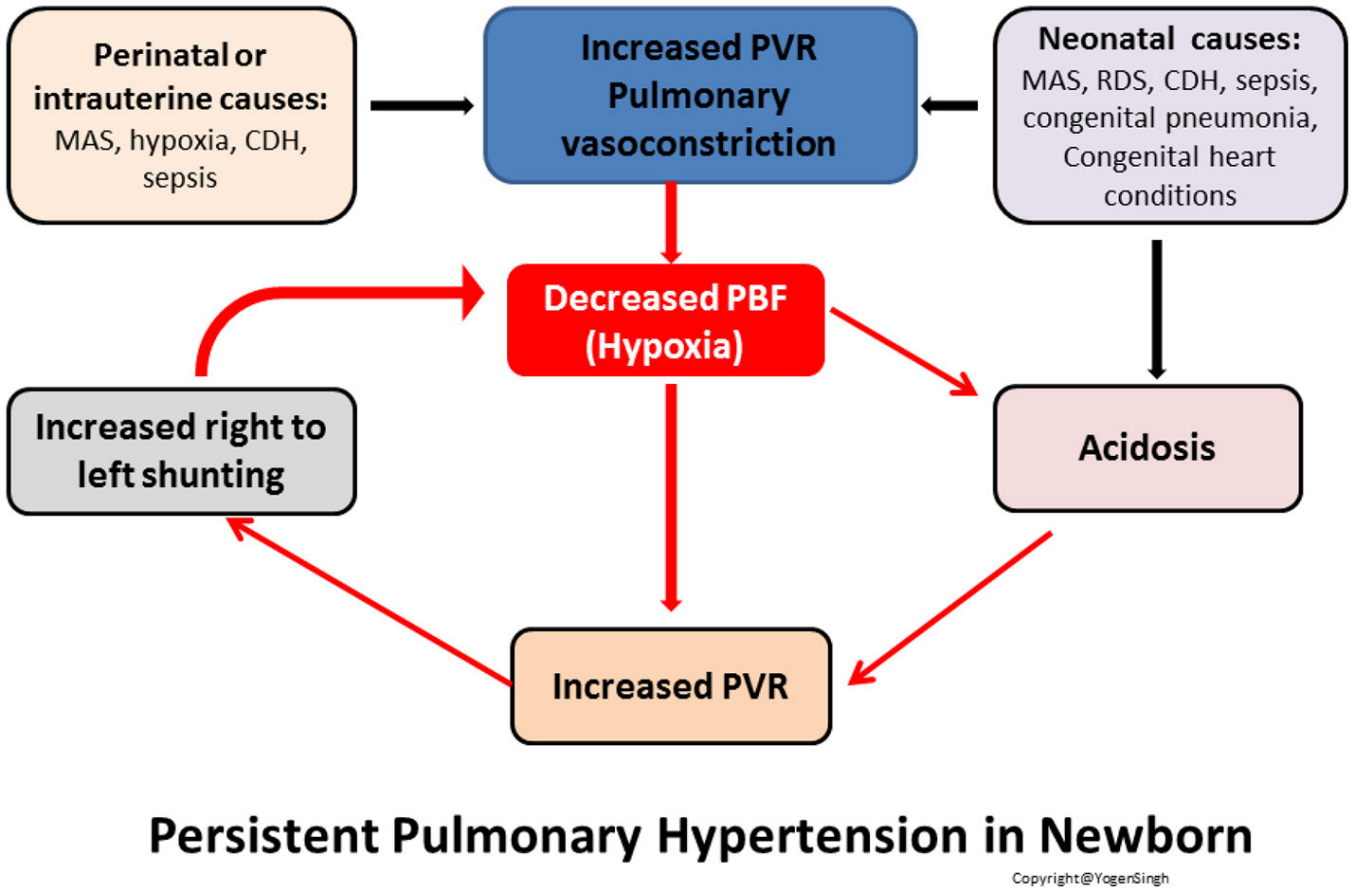
Pathophysiology of persistent pulmonary hypertension of newborn (PPHN). Acute pulmonary hypertension is the commonest adverse adaptation during cardiovascular transition after birth. PVR, pulmonary vascular resistance; PBF, pulmonary blood flow; MAS, meconium aspiration syndrome; RDS, respiratory distress syndrome; CDH, congenital diaphragmatic hernia.

The most immediate and adverse adaptation of transitional circulation occurs from persistent high PVR leading to continued right to left or bidirectional shunting across ductus arteriosus and/or via foramen ovale. Because of the increased PVR, PBF is decreased leading low oxygen saturation levels, increased oxygen requirement and ventilator support. This leads to decreased pulmonary venous return, and hence, decreased LV preload and low systemic blood flow (LV cardiac output). The decrease in perfusion of organs leads to increase in lactate, acidosis and hypoxia which are the most potent pulmonary vasoconstrictors. This further increases PVR, and it becomes a vicious cycle leading to acute pulmonary hypertension (commonly referred as persistent pulmonary hypertension of newborn or PPHN) (9, 10) (Figure 2).

The increase in RV afterload from persistently increased PVR (which is the hallmark of PPHN) results in right ventricular hypertrophy and/or dilatation and if this process continues then it may lead to RV dysfunction or RV failure. The right ventricle dysfunction and bowing of interventricular septum toward LV, due to increased RV pressure, further compromises LV preload and LV function leading to biventricular dysfunction (ventricular interdependence). The decreased LV preload and LV dysfunction result in low systemic blood pressure (hypotension) which often needs cardiovascular support (13). Bedside echocardiography can help in evaluating severity of pulmonary hypertension, assessment of cardiac function and hemodynamic assessment, and hence targeting the appropriate therapy to manage the pulmonary hypertension.

There is a high incidence of persistent PDA in the preterm infants, and its incidence is directly related to the gestation age—lower the gestation age higher the incidence of PDA (21). The shunt volume across DA increases as PVR falls (which may be slower than in the term infants because of respiratory distress, persistent metabolic acidosis and premature lung parenchyma leading to high PVR). Hence, there may be no clinical signs of PDA during the first few days after birth, and once left to right shunt becomes significant the signs and symptoms PDA appear (21). Moreover, the preterm myocardium has poor compliance and inherent diastolic dysfunction which makes it difficult to adapt to the extra shunt volume as compared to a term infant with normal myocardium (27).

The signs and symptoms of persistent PDA not only depend upon the size of ductus arteriosus but also on the magnitude of the shunt and the ability of myocardium to adapt to the extra shunt volume. This may explain why signs and symptoms vary in patients with a similar size of ductus arteriosus. Therefore, a thorough hemodynamic assessment is critical in evaluation of PDA and a bedside echocardiography can help in determining ductal characteristics, degree of pulmonary hyperperfusion (from increased pulmonary flow from left to right ductal shunt) and systemic hypoperfusion (below the ductal level) (28).

The other common adverse adaptation of transitional circulation occurs in the setting of perinatal asphyxia. Essentially similar mechanisms as explained above leads to adverse adaptation of transition leading to high PVR, poor cardiac function from direct cardiac compromise and often these infants have associated PPHN from associated or underlying conditions. The therapeutic hypothermia, which is a standard of care for infants with moderate to severe hypoxic-ischaemic encephalopathy, may further affect the transition—it leads to high PVR, high SVR, sinus bradycardia associated with low cardiac output and it may further deteriorate cardiac function from increased afterload (29, 30). A detailed mechanism is discussed in another article “advanced in diagnosis and management of shock” in Frontiers in Pediatrics (31).

## Transitional circulation in infants with congenital heart defects

As discussed above, the diagnosis of congenital heart defects (CHDs) may be challenging during transitional circulation. Similarly, the presence of congenital heart defect may affect the cardiovascular physiology and it's critical to understand this impact while managing infants with CHDs. A classification of the CHDs based upon the post-natal transition is summarized in Table 1.

**Table 1:** Classification of the congenital heart defects according to the post-natal adaptation.

**Types of CHD**		**Examples of CHD**
Duct-dependent CHD	Duct-dependent pulmonary circulation	Critical TOF
		PA/VSD
		PA/IVS
		Critical PS
		TA with PS/PA
		SV with PS/PA
		Severe Ebstein anomaly
	Duct-dependent systemic circulation	HLHS
		Critical AS
		Severe COA
		IAA
		Shone complex
		SV with AS/COA
	Poor mixing	TGA with IVS
Non duct-dependent CHD	Mild cyanotic CHD	TAPVR
		TOF
		TAC with mild PS
		TGA with VSD
		SV
	L-to-R shunt CHD	VSD
		PDA
		AVSD
		APW
		DORV
		TAC with no PS
		SV

*AS, aortic stenosis; ASD, atrial septal defect; APW, aorto-pulmonary window; AVSD, atrio-ventricular septal defect; CHD, congenital heart disease; COA, coarctation of aorta; DORV, double outlet right ventricle; HLHS, hypoplastic left heart disease; IAA, interrupted aortic arch; IVS, intact ventricular septum; PA, pulmonary atresia; PDA, patent ductus arteriosus; PS, pulmonary stenosis; SV, single ventricle; TA, tricuspid atresia; TAC, truncus arteriosus communis; TAPVR, total anomalous pulmonary venous return; TGA, transposition of the great arteries; TOF, Tetralogy of Fallot; VSD, ventricular septal defect*.

The hemodynamic changes due to CHDs are different during fetal life than those occurring after birth. Moreover, even after birth these changes are dynamic depending upon changes in SVR, PVR, shunt magnitude, and disease pathology. It is well known that early fetal obstruction of a ventricular outflow (right or left) tract may result in progressive underlined ventricular hypoplasia. Moreover, altered blood flow and oxygen content may affect pulmonary vascular bed and cerebral development. Most CHDs are well tolerated during the fetal life because oxygenation is primarily dependent upon placenta and fetal shunts allowing the fetal circulation to adapt. Nevertheless, CHD induce profound changes in the fetal hemodynamics that may alter post-natal adaptation (32).

The impact of CHD varies from benign with no repercussion after birth to serious with profound hemodynamic disturbances in the early post-natal life. Transposition of the great arteries (TGA), right and left heart obstructive lesions, and obstructed total anomalous pulmonary venous return (TAPVR) are serious conditions carrying a high early mortality. Those CHDs are poorly tolerated during the transitional phase where the function of oxygenation is transferred from the placenta to the lungs with subsequent decline in PVR, increase in SVR, closure of the ductus arteriosus and closure of the foramen ovale. When untreated, they can progress to severe acidosis, shock and/or cyanosis (32, 33).

### Duct-dependent congenital heart defects

#### Duct-dependent systemic circulation

**Figure 3:**
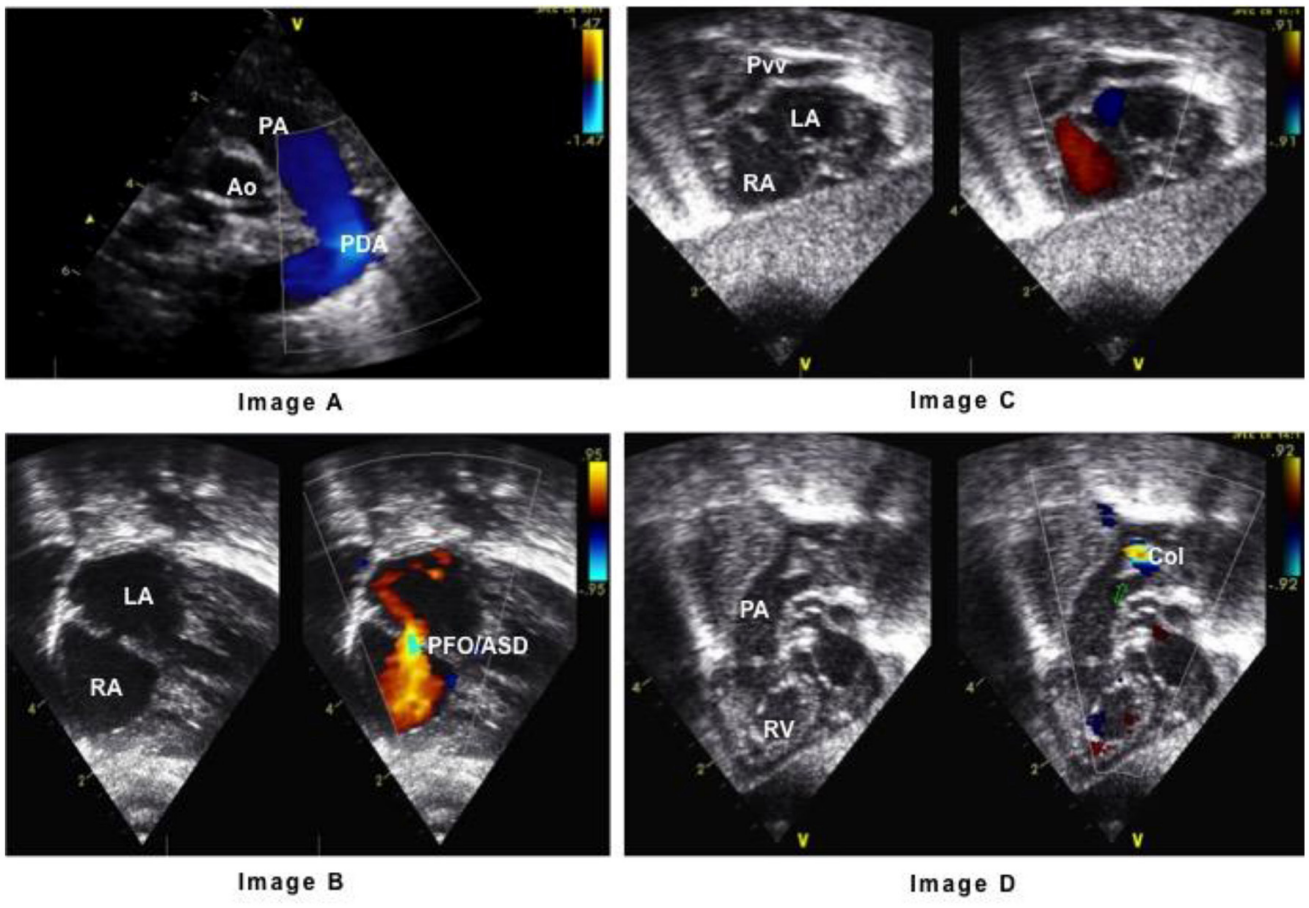
Echocardiography parasternal short axis view in systemic duct-dependent circulation with right-to-let shunt across the patent ductus arteriosus (Image **A**). Echocardiography subcostal view in transposition of the great arteries (TGA) with left-to-right interatrial mixing between the pulmonary and systemic circulation (Image **B**). Echocardiography subcostal view in total anomalous pulmonary venous return (TAPVR) with right-to-left interatrial mixing of the pulmonary and systemic circulation (Image **C**). The level of obstruction of pulmonary venous collector is usually at the branch pulmonary arteries (arrow, Image **D**). Ao, aorte; ASD, atrial septal defect; Col, pulmonary venous collector; LA, left atrium; PA, pulmonary artery; PFO, patent foramen ovale; PDA, patent ductus arteriosus; Pvv, pulmonary veins; RA, right atrium.

Left heart obstructive lesions require an open ductus arteriosus to maintain the systemic circulation. When the ductus arteriosus closes, the SVR decreases, the systemic perfusion falls, the pulses become weak and the newborn develops progressive acidosis and shock (34). Prostaglandin E1/E2 infusion is necessary to maintain the ductus open and allow for right-to-left shunting (Figure 3, image A).

#### Duct-dependent pulmonary circulation

Right heart obstructive lesions require an open ductus arteriosus to maintain pulmonary circulation. When the ductus arteriosus closes, the pulmonary perfusion falls and the newborn develops progressive cyanosis with no response to oxygen administration. The administration of oxygen will further induce constriction of the ductus arteriosus. Because the fetal physiology is adapted to chronic hypoxia, those babies can tolerate cyanosis better than the older child until acidosis develops (34). Prostaglandin E1/E2 infusion will allow the ductus to stay open with left-to-right shunt.

#### Transposition of the great arteries (TGA)

Transposition of the great arteries with intact ventricular septum (TGA/IVS) can be considered as a duct-dependent lesion, although an atrial septal defect (ASD) or a patent foramen ovale (PFO) is crucial to allow for mixing between the pulmonary and systemic circulations (Figure 3, image B). Nevertheless, when neonatal adaptation is smooth and is followed by a fall in PVR and pulmonary arterial pressure (PAP), the PDA allows for left-to-right shunt and for increased PBF, increased pulmonary venous return and increased preload of the left atrium. The subsequent increase in left atrial pressure is crucial for left-to-right interatrial shunting and mixing between the pulmonary venous and systemic venous returns. When neonatal adaptation is poor with subsequent hypoxemia, the fall in PVR is delayed, PBF is reduced and interatrial mixing is compromised. These infants need emergency atrial septostomy (Rashkind maneuver). It has been shown that fetuses with restriction of the foramen ovale, constriction of the ductus arteriosus, and bidirectional flow across the ductus arteriosus during the 3rd trimester of pregnancy are at increased risk for early post-natal severe hypoxemia (32, 35).

### Congenital heart defects with mild cyanosis

TAPVR requires mixing of the pulmonary and systemic circulation at the atrial level (right-to-left interatrial shunt as seen in Figure 3, image C) allowing for moderate desaturation. In patients with obstruction of the pulmonary venous flow (Figure 3, image D), there will be progressive decrease in pulmonary venous return to the systemic vein and to the right atrium with subsequent progressive cyanosis. The increase in pulmonary venous pressure will cause pulmonary edema and respiratory distress, mimicking parenchymal lung disease, and these infants will not respond to oxygen supplementation. Prostaglandin infusion is not helpful and in fact it may lead to further facilitate left-to-right ductal shunt, increased PBF, pulmonary edema and heart failure. The only treatment is early primary repair (32).

CHD with moderate right outflow tract obstruction [Tetralogy of Fallot (TOF), truncus arteriosus communis (TAC)] and right-to-left shunt through a ventricular septal defect (VSD) may present mild cyanosis. The degree of cyanosis will depend on the severity of the pulmonary stenosis. TGA with VSD can present with mild cyanosis, and it is usually not a duct-dependent lesion due to the mixing of the pulmonary and systemic circulations across the VSD. If the VSD is small and the foramen ovale is restrictive or intact, prostaglandin E1/E2 infusion may be necessary; and an urgent atrial septostomy should be performed (32, 34).

### Transitional circulation in infants with CHDs and left-to-right shunts

The infants with systemic-to-pulmonary communications (ventricular septal defect, PDA, aorto-pulmonary window) are usually unremarkable soon after birth because the fall in PVR is progressive, and it takes at least 2 weeks to be complete, even longer in babies with those CHDs. In the early post-natal period, the amount of left-to-right shunt is low and does not cause any symptoms. With the progressive fall in PVR, the amount of left-to-right shunt will increase according to the size of the systemic-to-pulmonary communication, and it results in increased PBF. The left ventricular output increases accordingly to maintain adequate systemic blood flow. The increase in left atrial pressure may lead to pulmonary edema, respiratory distress and poor weight gain (35, 36).

## Echocardiographic assessment during transition circulation and its adverse adaptation

Bedside echocardiography may play an important role in understanding the normal cardiovascular transition physiology and its adverse adaptation. The echocardiographic assessment during a normal smooth transition process, and during the most common adverse adaptations (persistent pulmonary hypertension, prematurity, and perinatal asphyxia) are discussed below:

### Echocardiography during normal transition physiology

**Figure 4:**
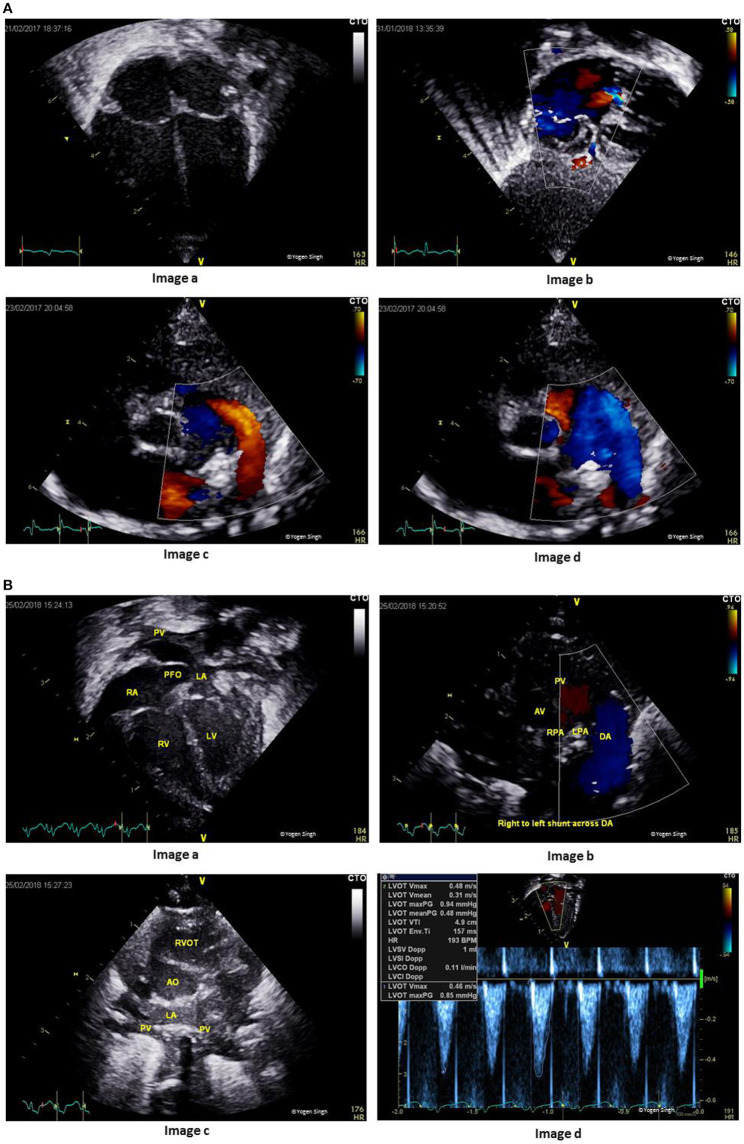
**(A)** Echocardiographic assessment during transitional circulation. Image “a” shows mild right ventricular dominance (physiological); Image “b” shows bidirectional shunt across foramen ovale; Image “c” shows left to right shunt across ductus arteriosus; and Image “d” shows right to left shunt across ductus arteriosus. Image “c” and image “d” are still frames from bidirectional shunt during early transitional circulation (which may be normal as compared to adverse adaptation in Figure 1C). **(B)** Echocardiographic assessment during adverse adaptation of transitional circulation in a preterm infant with sepsis at 8 h of age. Image “a” shows dilatation of right side of the heart in apical 4-chamber view with inter-atrial septum bowed toward left atrium (LA); Image “b” shows pure right to left shunt across ductus arteriosus (DA) because of high pulmonary vascular resistance (PVR) and low systemic vascular resistance (SVR); Image “c” shows small LA from decreased pulmonary return (decreased pulmonary blood flow due to high PVR and right to left shunt across DA; and Image “d” shows decreased left ventricle cardiac output (from decreased LV preload and impaired LV function).

The echocardiographic assessment during normal transition physiology may show presence of PDA, patent foramen ovale and right ventricular dominance if it is performed soon after birth or in presence of delayed adaptation to transition (Figure 4).

The shunt across foramen ovale is either left to right or bidirectional but never purely right to left. A pure right to left shunt across foramen ovale should raise suspicion of underlying CHDs (such as total anomalous pulmonary venous drainage or tricuspid atresia) (13, 35, 36).

The shunt across ductus arteriosus changes to bidirectional or left to right soon after birth (Figures 4A,B). A pure right to left ductal shunt should be carefully evaluated to rule out severe PPHN (often these infants are sick needing significant cardiorespiratory support) or underlying duct dependent CHDs (such as interrupted aortic arch). Mild tricuspid regurgitation with a velocity <2.6 m/s (gradient between RV and RA under 25 mmHg) is normal and often seen during normal transition.

### Echocardiography during adverse adaptation of transitional circulation

The echocardiographic assessment during adverse adaptation should include a thorough structural assessment to rule out underlying congenital heart defect, assessment of cardiac function and hemodynamic evaluation. In conjunction with other clinical parameters, a bedside functional echocardiography may provide immediate anatomical and physiological information in real time to help in understanding the pathophysiology leading to adverse adaptation and in targeting the specific intervention based upon altered physiology. While the detailed assessment of the individual specific conditions is discussed in other TINEC research articles published in Frontiers in Pediatrics, an approach to echocardiographic assessment to common conditions associated with adverse adaptation of transitional circulation is discussed below:

#### Echocardiographic assessment in persistent pulmonary hypertension

As discussed above, increased PVR is the hallmark of altered transition physiology in persistent pulmonary hypertension. The direct assessment of PVR may be difficult to assess on bedside echocardiography but a detailed assessment of the severity of pulmonary hypertension, cardiac function and hemodynamic assessment may be helpful in managing the adverse adaptation in patients with high PVR or pulmonary hypertension.

**Figure 5:**
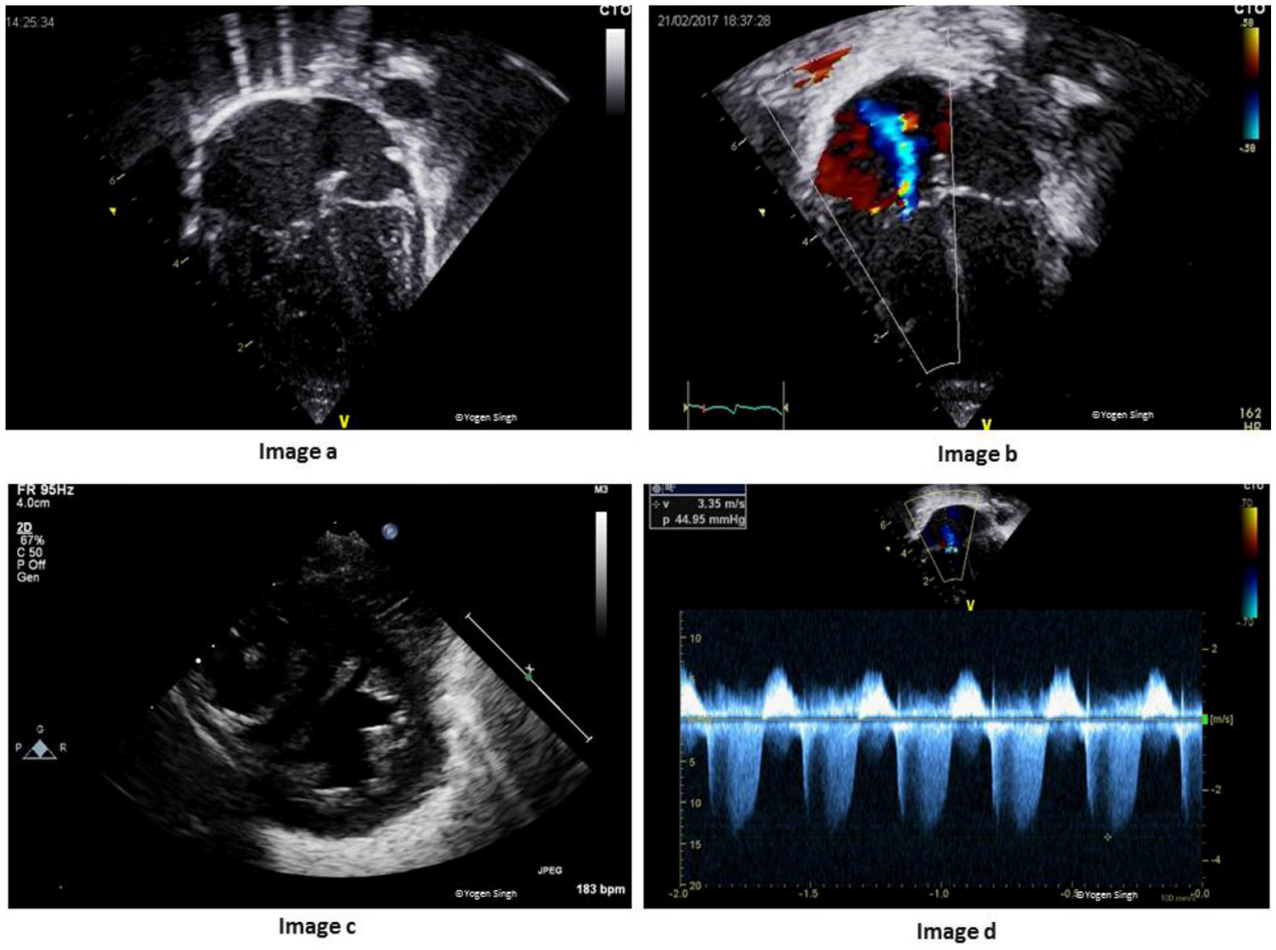
Echocardiographic assessment of pulmonary hypertension in adverse adaptation of transitional circulation. Image “**a**” shows right ventricular hypertrophy and mild dilatation in apical 4-chamber view; Image “**b**” shows tricuspid jet on color flow mapping; Image “**c**” shows hypertrophy of right ventricular and bowing of interventricular septum in parasternal short axis view; and Image “**d**” shows Doppler assessment of tricuspid regurgitation (TR) to assesses pressure gradient between right ventricle and right atrium. Pulmonary artery systolic pressure can be estimated by adding right atrial pressure to the pressure gradient measured by TR velocity.

A persistent high PVR (RV afterload) may lead to right ventricular hypertrophy or even RV dysfunction with or without RV dilatation. The severity of pulmonary hypertension is best assessed by measuring pulmonary artery pressure. It can also be indirectly assessed by assessing interventricular septum and LV shape. The shunt across PDA may be bidirectional or purely right to left depending upon the pulmonary artery pressure in relation to systemic pressure. The shunt across foramen ovale is often bidirectional. The RV cardiac output may be decreased in presence of high PVR and RV dysfunction, and LV cardiac output may be decreased from decreased LV preload (decreased pulmonary venous return) and LV dysfunction (37). A thorough assessment of adverse adaptation in persistent pulmonary hypertension (13) may include (Figure 5):

- Recognition of RV hypertrophy with or without RV dilatation
- Estimation of pulmonary artery pressure
- Assessment of shunt across ductus arteriosus and foramen ovale
- Assessment of interventricular septum and LV shape
- Evaluation of RV and LV function
- Cardiac filling (preload assessment)
- Measurement of RV and LV cardiac output.

#### Echocardiographic assessment of adverse adaptation in premature infants

Like the term infants, most of the mature preterm infants may have a smooth transition. However, this process may be more challenging for the extremely low birth weight (ELBW) infants under 28 weeks of gestation. There are certain specific factors related to prematurity that may affect the transition process in preterm infants: (1) higher incidence of persistent PDA and foramen ovale; shunting across PDA may play an important role in the disease pathology; (2) impaired myocardial performance due to immature myocardium possessing inefficient contractile mechanism leading to inherited impaired systolic and diastolic cardiac function in ELBW infants; (3) faster heart rate with possibly increased cardiac demand and less time spent in diastole limiting the ability to increase stroke output; and (4) higher incidence of respiratory conditions needing mechanical ventilation and affecting cardiovascular dynamics (6, 38, 39). All these mechanisms limit the ability of preterm myocardium to cope with adverse loading conditions (increased afterload from persistent high PVR or decreased/increased preload). The studies report that premature myocardium may lack adequate adrenergic innervation, and they may have under underdeveloped hypothalamic–pituitary–adrenal axis. Hence, they may not only have limited abilities to increase cardiac output during adverse adaptation but they may also show inadequate response to stressful situations (such as shock and sepsis) and a poor response to inotropes (40, 41).

**Figure 6:**
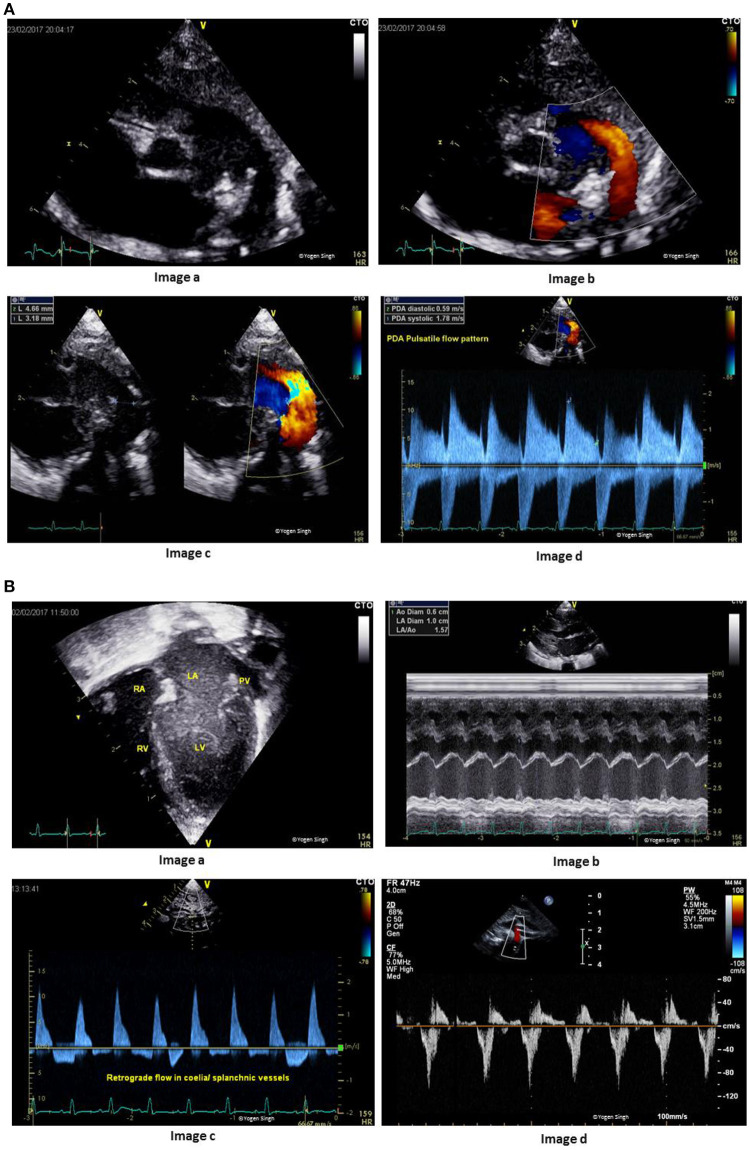
**(A)** Assessment of patent ductus arteriosus (PDA). Image “a” shows PDA on 2D imaging; Image “b” shows PDA on color flow mapping; Image “c” assessment of PDA size on 2D and color flow mapping; and Image “d” shows pulsatile flow pattern on Doppler assessment. **(B)** Hemodynamic assessment of PDA for assessing pulmonary hyperperfusion and systemic hypoperfusion. Image “a” shows volume overloading left side of the heart in apical 4-chamber view; Image “b” shows measurement of left atrium to aorta ratio (LA:Ao ratio); Image “c” shows retrograde flow in splanchnic vessels; and Image “d” retrograde flow in descending aorta (“steal phenomenon”) in a hemodynamically significant large PDA.

Therefore, understanding the cardiovascular physiology may be even more important in ELBW infants while making a careful decision regarding fluid therapy and inotropes during adverse adaptation. A persistent large left to right shunt across PDA in ELBW infants may lead to significant hemodynamic changes: systemic hypoperfusion (below the ductal level) and pulmonary hyperperfusion. The summary of the echocardiographic assessment of PDA and its hemodynamic evaluation is demonstrated in Figures 6A,B. A detailed echocardiographic evaluation is described in another article under the TINEC theme published in Frontiers in Pediatrics (42).

#### Echocardiographic assessment of adverse adaptation due to perinatal asphyxia

Perinatal asphyxia refers to oxygen deprivation before or around the time of birth and it may results in hypoxia-ischemia (HI) induced brain damage or hypoxic-ischemic encephalopathy (HIE). The cardiovascular instability is common in patients with HIE and the causes are multifactorial. In fact, transient myocardial ischemia and myocardial dysfunction occurs in two-thirds of the infants with moderate to severe perinatal asphyxia (43, 44).

The effects of perinatal asphyxia on the cardiovascular transition are complex. Myocardial ischaemia may be a direct effect of perinatal asphyxia or it may be further caused by the ongoing redistribution of blood flow leading to reduced myocardial perfusion, especially in the sub-endocardial tissue and papillary muscles (43–45). This leads to myocardial dysfunction. The infants with perinatal asphyxia often have multi-organ failure and a persistent high PVR leading to PPHN is a common association. Hypoxia and acidosis from poor cardiac perfusion further worsens pulmonary hypertension by increasing PVR.

In addition, therapeutic hypothermia also increases PVR and SVR (46), which can have a further unfavorable effect on the already compromised myocardial function. This can lead to poor cardiac output and impaired end organ perfusion, and hence worsening of acidosis. Moreover, neonates with perinatal asphyxia often have associated morbidities, such as sepsis, abnormal placental physiology and MAS, affecting hemodynamics and cardiac function during the transition process.

Bedside echocardiography can help in assessing the cardiac function and hemodynamic changes in real time. The assessment of preload, cardiac output and cardiac function may help in making a logical choice of therapy in patients with moderate to severe HIE to optimize the end-organ blood flow.

#### Echocardiographic assessment of adverse adaptation due to congenital heart defects

As discussed above, the infants with CHDs may have an effect on the transitional physiology such as increased left to right shunt may lead to increased PBF. When shunt is significant it may contribute to worsening of respiratory symptoms such as tachypnoea and increased work of breathing requiring oxygen and/or respiratory support. A persistently increased PBF may contribute to development of pulmonary hypertension in children (7, 9).

Similarly, hypoxia from decreased blood flow may affect the normal transition process. These children often have associated conditions leading to decreased blood flow (such as right ventricular outflow obstruction) which may lead to ventricular hypertrophy, impaired diastolic and systolic functions. The effects of the CHDs can be even more complex to understand in presence of multiple defects or complex association with varying effects on cardiovascular physiology (36, 47). A condition specific detailed description affecting the cardiovascular physiology during neonatal period is beyond the scope of this article emphasizing on the general principles of transition and its adverse adaptation.

#### Echocardiographic assessment of adverse adaptation due to other neonatal conditions

The other neonatal conditions may have an impact on the cardiovascular transitional process such as neonatal sepsis. It may have direct effect on the myocardial performance leading to cardiac dysfunction or may affect the vasomotor permeability leading the change in SVR. Unlike adults with warm septic shock leading to drop in SVR, neonates may have a complex response to shock. SVR may be increased, especially during early sepsis (cold shock) or it may be decreased (warm shock). The studies have reported that sepsis may have deleterious effect on the myocardial function—both direct effect on myocardium or via cytokine induced pathway. The troponin levels in non-survivors of neonatal sepsis have been reported significantly higher than survivors (29, 48). Similarly, other neonatal conditions such as necrotizing colitis and intraventricular hemorrhage may affect the normal transition process.

## Summary

Transitional physiology is complex and it may be altered by multifactorial causes. The adverse adaptation of transition physiology may lead to persistent pulmonary hypertension, pulmonary hyper- or hypo-perfusion and systemic hypoperfusion. In conjunction with a meticulous clinical examination and routine bedside monitoring, the use of new technologies such as functional echocardiography, non-invasive assessment of cardiac output and end-organ perfusion assessment by using near infra-red spectroscopy (NIRS) may provide invaluable information in the infants with altered transitional physiology. While the role of other techniques is being established, bedside echocardiography may provide anatomical and physiological information in real time helping to make judicious clinical decisions in the intensive care setting.

## Author contributions

CT is a pediatric cardiologist trained at the University Hospital of Geneva, Switzerland and in Denver, Colorado, USA. CT has been working as an attending pediatric cardiologist at the Children's Hospital of Geneva (HUG) until recently and is now working in the Pediatric Center at Clinique des Grangettes, Switzerland. YS is a consultant in neonatology and pediatric cardiology at Addenbrooke's Hospital in Cambridge, UK and he is a course director for echocardiography in Cambridge. CT and YS have special interest in functional echocardiography and hemodynamic assessment in children. They both belong to the NPE (Neonatologist Performed Echocardiography) group form the ESPR (European Society of Pediatric Research) and are chairing the POCUS (Point-of-Care Ultrasound) working group from the ESPNIC (European Society of Pediatric and Neonatal Intensive Care). They belong to the organizing committee of TINEC (Training in Intensive care and Neonatal Echocardiography), a course on functional echocardiography taking place every year in Switzerland since 2016.

### Conflict of interest statement

The authors declare that the research was conducted in the absence of any commercial or financial relationships that could be construed as a potential conflict of interest.
