# Corrigendum: Echocardiographic Evaluation of Transitional Circulation for the Neonatologists

**DOI:** 10.3389/fped.2020.600496

**Published:** 2020-11-17

**Authors:** Yogen Singh, Cécile Tissot

**Affiliations:** ^1^Consultant in Neonatology and Pediatric Cardiology, Cambridge University Hospitals NHS Foundation Trust, Cambridge, United Kingdom; ^2^School of Clinical Medicine, University of Cambridge, Cambridge, United Kingdom; ^3^Pediatric Cardiologist, Centre de Pediatrie, Clinique des Grangettes, Chêne-Bougeries, Geneva, Switzerland

**Keywords:** transitional circulation, adverse adaptation of transitional circulation, persistent pulmonary hypertension, hemodynamic assessment, fetal to neonatal transition, patent ductus arteriosus, PDA, PPHN

In the original article, there was a mistake in ^**^Table 1^**^ as published. ^**^Duct-dependent systemic circulation and Duct-dependent pulmonary circulation terms were interchanged^**^. The corrected ^**^Table 1^**^ appears below.

**Table 1:** Classification of the congenital heart defects according to the post-natal adaptation.

**Types of CHD**		**Examples of CHD**
Duct-dependent CHD	Duct-dependent pulmonary circulation	Critical TOF
		PA/VSD
		PA/IVS
		Critical PS
		TA with PS/PA
		SV with PS/PA
		Severe Ebstein anomaly
	Duct-dependent systemic circulation	HLHS
		Critical AS
		Severe COA
		IAA
		Shone complex
		SV with AS/COA
	Poor mixing	TGA with IVS
Non duct-dependent CHD	Mild cyanotic CHD	TAPVR
		TOF
		TAC with mild PS
		TGA with VSD
		SV
	L-to-R shunt CHD	VSD
		PDA
		AVSD
		APW
		DORV
		TAC with no PS
		SV

*AS, aortic stenosis; ASD, atrial septal defect; APW, aorto-pulmonary window; AVSD, atrio-ventricular septal defect; CHD, congenital heart disease; COA, coarctation of aorta; DORV, double outlet right ventricle; HLHS, hypoplastic left heart disease; IAA, interrupted aortic arch; IVS, intact ventricular septum; PA, pulmonary atresia; PDA, patent ductus arteriosus; PS, pulmonary stenosis; SV, single ventricle; TA, tricuspid atresia; TAC, truncus arteriosus communis; TAPVR, total anomalous pulmonary venous return; TGA, transposition of the great arteries; TOF, Tetralogy of Fallot; VSD, ventricular septal defect*.

The authors apologize for this error and state that this does not change the scientific conclusions of the article in any way. The original article has been updated.

